# *Astragalus membranaceus* Root Extract Improves T Cell Immunity in CTX-Immunosuppressed Mice and Is Associated with Hypermethylation of the *Cpt1a* Locus

**DOI:** 10.3390/ijms27177746

**Published:** 2026-08-29

**Authors:** Minqiang Gao, Tong Sun, Weihua Zhao, Jiande Li, Yanjie Dong, Jiangyuan Han

**Affiliations:** 1 College of Life Science and Technology, Gansu Agricultural University, Lanzhou 730070, China; 2 College of Life Sciences, Northwest Normal University, Lanzhou 730070, China; 3 West China School of Medicine, Sichuan University, Chengdu 610041, China

**Keywords:** *Astragalus membranaceus*, immunosuppression, DNA methylation, *Cpt1a*

## Abstract

*Astragalus membranaceus* (AM) has been reported to support immune function, but its epigenetic mechanism on immune restoration of cyclophosphamide (CTX)-induced immunosuppression remains unclear. To evaluate the recovery of immune function, we assessed the injury of splenic pathology, cytokines levels in serum, proportional changes in CD4^+^ and CD8^+^ T cells, and the recall response of Hspx-specific T cell. In addition, to define the underlying epigenetic regulatory mechanism, we measured the expression levels of *DNA methyltransferases* (*Dnmt1*, *Dnmt3a* and *Dnmt3b*) and the methylation status of the *Carnitine palmitoyltransferase 1A* (*Cpt1a*) gene. The results showed that the crude extract of AM repaired the immunosuppression induced by CTX. This is reflected in the alleviation of splenic pathological injury, the elevated proportions of CD4^+^ and CD8^+^ T cells, and the increased levels of serum IL-2 and IFN-γ. The crude extract of AM also enhanced the recall response of Hspx-specific CD8^+^ T cells; that is, the frequencies of the resulting IL-2^+^ and IFN-γ^+^ cells were significantly higher than those in the CTX + Vaccine group. All these results indicate that the adaptive immune response induced by the crude extract of AM is significantly enhanced. On the other hand, AM crude extract reversed the CTX-induced downregulation of *Dnmt3a* mRNA and restored CpG methylation of *Cpt1a* gene to a level comparable to the vaccine group at molecular level. Consistent with the elevated CpG methylation, CTX-induced upregulation of *Cpt1a* mRNA was also reduced. These findings reveal that AM extract enhances the antigen-specific functional recall responses of CD8^+^ T cells in immunosuppressed mice were associated with *Cpt1a* gene methylation, thereby establishing a translational link between traditional herbal medicine and modern epigenetics.

## 1. Introduction

Cyclophosphamide (CTX) is a classic alkylating agent that exerts anticancer effects through DNA cross-linking [1,2,3]. Its non-selective cytotoxicity depletes CD4^+^ and CD8^+^ T cells, B cells, and dendritic cells, leading to prolonged lymphocytopenia and bone marrow suppression [4,5]. This immune dysfunction will increase the risk of opportunistic infections and may impair the efficacy of subsequent immunotherapy [6,7]. Current immunomodulators for cancer supportive care (e.g., G-CSF, thymosin α1, levamisole) have notable limitations, including high cost, mandatory parenteral administration, and variable clinical efficacy [8,9]. Safe, cost-effective, orally available alternatives remain an unmet clinical need. Thus, identifying low-toxicity botanical agents capable of restoring multifaceted immune function represents a promising strategy for cancer supportive therapy.

*Astragalus membranaceus* (AM), a core medicinal herb in traditional Chinese medicine, exerts well-documented immunomodulatory activities [10]. Modern pharmacological studies have identified several bioactive components in AM extract, including astragalosides, polysaccharides, and flavonoids, which can promote lymphocyte proliferation and regulate cytokine production [11,12,13]. However, under the condition of chemotherapy-induced immunosuppression, the specific mechanism by which AM restores T cell functions remains largely unelucidated.

Recent studies in the field of immunometabolism have demonstrated that lymphocytes undergo rapid metabolic reprogramming during the processes of activation and proliferation [14,15]. Fatty acid β-oxidation (FAO), which is partially regulated by the rate-limiting enzyme *Carnitine palmitoyltransferase 1a* (*Cpt1a*), contributes to the long-term survival and recall response of memory T cells under physiological conditions [16,17]. However, the role of *Cpt1a* is more complex in the tumor microenvironment. Tumor cells upregulate FAO to support their proliferation and immune evasion [18]. Furthermore, CPT1A-mediated FAO in plasmacytoid dendritic cells has been shown to promote immunosuppression. Conversely, *Cpt1a* inhibition can alleviate this suppression and enhance the function of CD8^+^ T cell [19]. Intriguingly, emerging evidence suggests that the transcriptional regulation of *Cpt1a* is governed by epigenetic modifications, particularly DNA methylation, which determines its expression in various pathological contexts [20]. These findings indicate that although physiological levels of *Cpt1a* activity are beneficial to the maintenance of memory T cell, its aberrant upregulation in the tumor microenvironment may lead to immune dysfunction. Given that DNA methylation is a stable epigenetic marker that is frequently dysregulated after chemotherapeutic stress, little is still known about how CTX reshapes the epigenetic landscape that controls *Cpt1a* expression in surviving T cells.

Given that the immunomodulatory effects of AM have been well-documented, and the regulatory role of *Cpt1a* in T cell immunity has attracted increasing attention, this study investigated whether AM alleviates CTX-induced immunosuppression by regulating *Cpt1a*. We established a CTX-induced immunosuppressed mouse model to evaluate the immunostimulatory effect of AM extract, explore its impact on the long-term immune response, and further elucidate the underlying mechanism of DNA methylation-regulated *Cpt1a* expression in mediating these effects.

## 2. Results

### 2.1. Identification of Chemical Constituents in AM Root Extract

**Figure 1:**
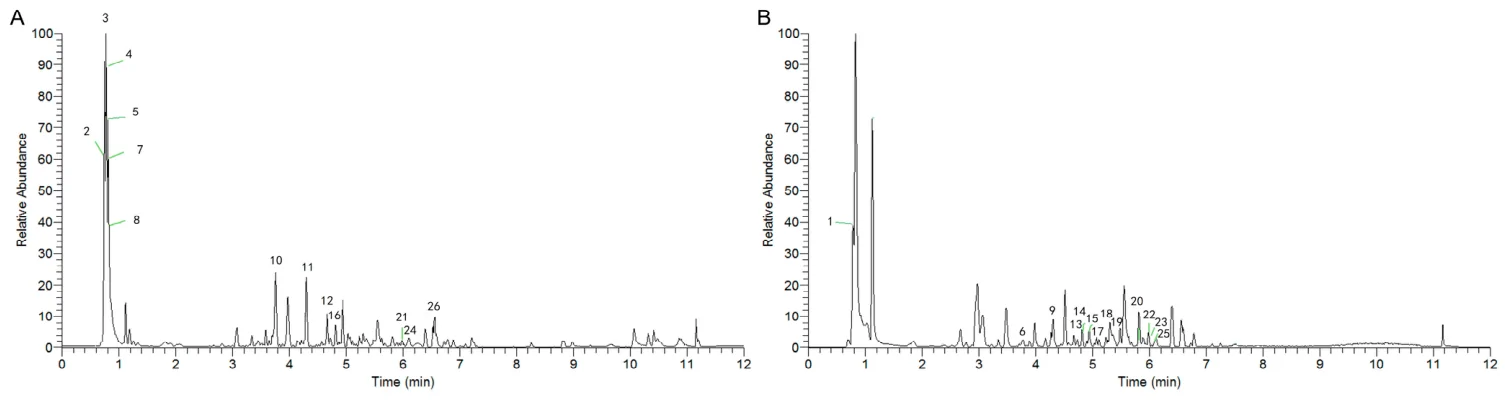
UPLC-MS total ion chromatogram of AM. (**A**) Positive ion mode and (**B**) negative ion mode.

Ultra-performance liquid chromatography coupled with tandem mass spectrometry (UPLC-MS) was applied to characterize the chemical constituents of the 70% ethanol extract from AM crude root decoction. Compounds were putatively identified by matching their retention time, accurate mass (mass tolerance ≤ 5 ppm), and MS/MS fragmentation patterns against HMDB and KEGG databases. A total of 26 compounds were identified, including 12 saponins (Astragaloside I, II, IV, VII; Asparasaponin II; Soyasaponin I, II, III; Astraikokioside I; Pseudoprotodioscin; and two additional peaks corresponding to Astragaloside IV and Astragaloside II, which were presumed to be different adducts or isomers), 5 flavonoids (Isovitexin 2″-O-β-d-glucoside, Isomucronulatol 7-O-glucoside, Mucronulatol, Quercetin 3-O-rhamnoside 7-O-glucoside, and Isoorientin 6″-O-α-l-arabinoside), 3 alkaloids (Pipecolic acid, Nicotianamine, and Feruloylagmatine), and 6 other compounds (L-aspartic acid, 4-hydroxy-3-methylbenzaldehyde, DL-arginine, Syringin, Vanillyl alcohol, and 4-hydroxybenzoic acid). The total ion chromatogram (TIC) is shown in Figure 1, and the detailed identification results are summarized in Table 1.

### 2.2. AM Crude Root Extract Attenuated CTX-Induced Immunosuppression in Mice

**Figure 2:**
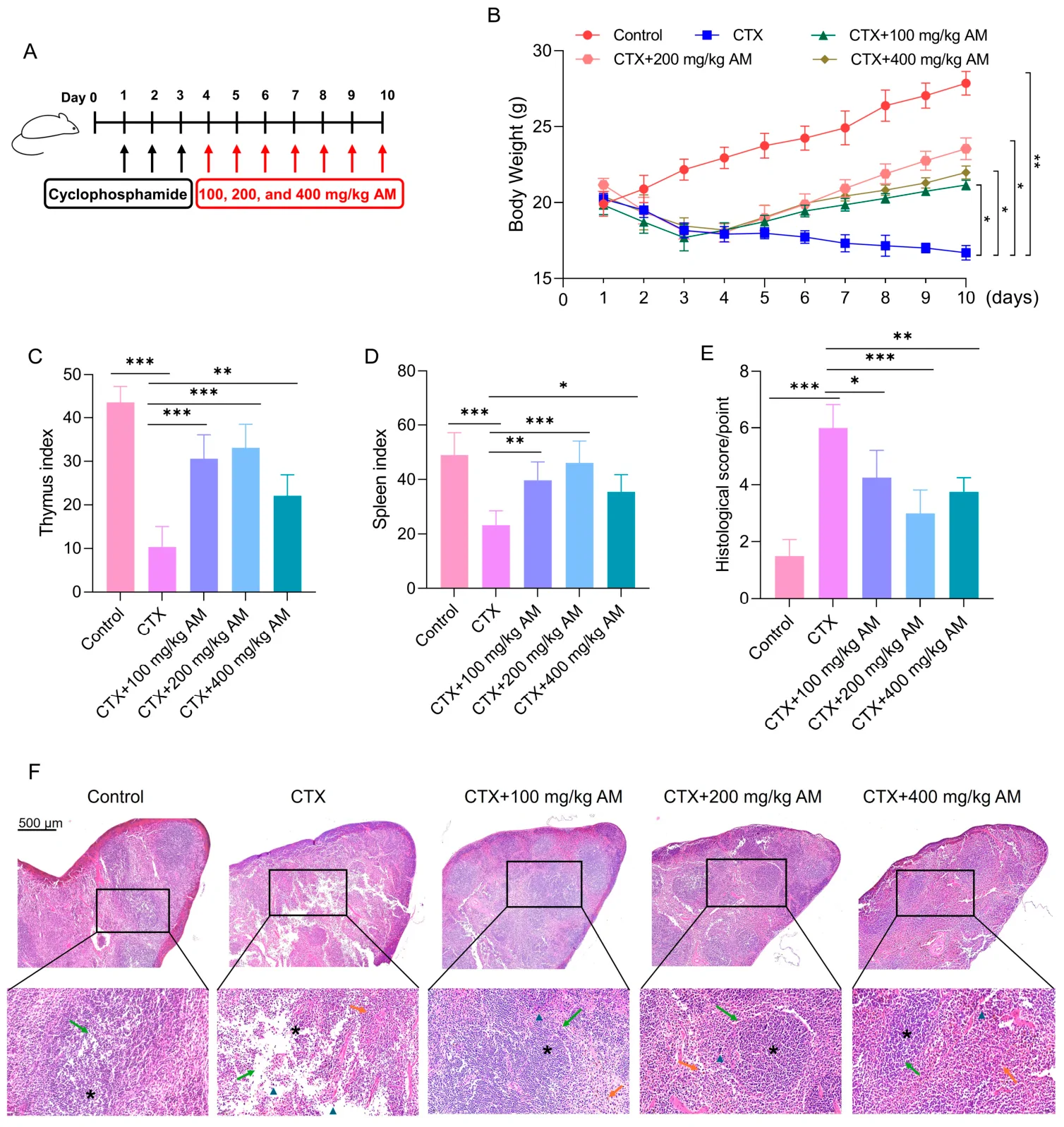
Effect of AM extract on CTX-induced immunosuppression in mice. (**A**) Schematic diagram of experimental schedule; (**B**) Body weight changes during the 7-day treatment period. (**C**,**D**) The indices of (**C**) thymus and (**D**) spleen. (**E**) Histological scores of pathological analysis of mouse spleen. (**F**) Hematoxylin-eosin (H&E) staining of the spleen. Asterisks indicate splenic corpuscles, green arrows mark the boundary of the white pulp, orange arrows denote blood cell stasis, and blue arrows indicate cell necrosis; scale bar represents 500 μm. Data are mean ± SD (*n* = 4). Statistical significance was determined by one-way ANOVA followed by Tukey’s post hoc test. * *p* < 0.05, ** *p* < 0.01, *** *p* < 0.001.

To further explore its immunoregulatory effects, the crude root extract of AM was evaluated in a mouse model of CTX-triggered immunosuppression. Body weight alterations of mice were recorded daily throughout the study period. Compared with the CTX-treated model group, mice in the 100, 200, and 400 mg/kg AM treatment groups showed significantly higher body weight gain throughout the 7-day intervention period (Figure 2B, *p* < 0.05). Meanwhile, the thymus index (Figure 2C, *p* < 0.001 for CTX vs. CTX + 100 and CTX + 200 mg/kg AM; *p* < 0.01 for CTX vs. CTX + 400 mg/kg AM) and splenic index (Figure 2D, *p* < 0.01 for CTX vs. CTX + 100; *p* < 0.001 for CTX vs. CTX + 200 mg/kg AM; *p* < 0.05 for CTX vs. CTX + 400 mg/kg AM) were also increased in all AM treatment. Histopathological examination of the spleen tissues further validated the above observations. As illustrated in Figure 2F, the spleens from the healthy control mice presented clear demarcation between red pulp and white pulp, along with densely distributed lymphocytes. In contrast, CTX treatment caused severe disruption of splenic architecture, including indistinct white pulp, vascular congestion, blurred boundaries, atrophic lymphoid nodules, and marked lymphocyte depletion. Histological scoring revealed that all AM extract treatment groups significantly alleviated splenic pathological damage compared with the CTX group. A notable protective effect was observed in the 200 mg/kg AM group, where the splenic structure nearly improved to the intact level of the control group (Figure 2E, *p* < 0.001). The observed improvements included reduced vascular congestion and necrosis, clearer red/white pulp demarcation, and more distinct lymphoid nodules.

### 2.3. AM Extract Increases CD4^+^/CD8^+^ T Cells and Serum Cytokines in Immunosuppressed Mice

**Figure 3:**
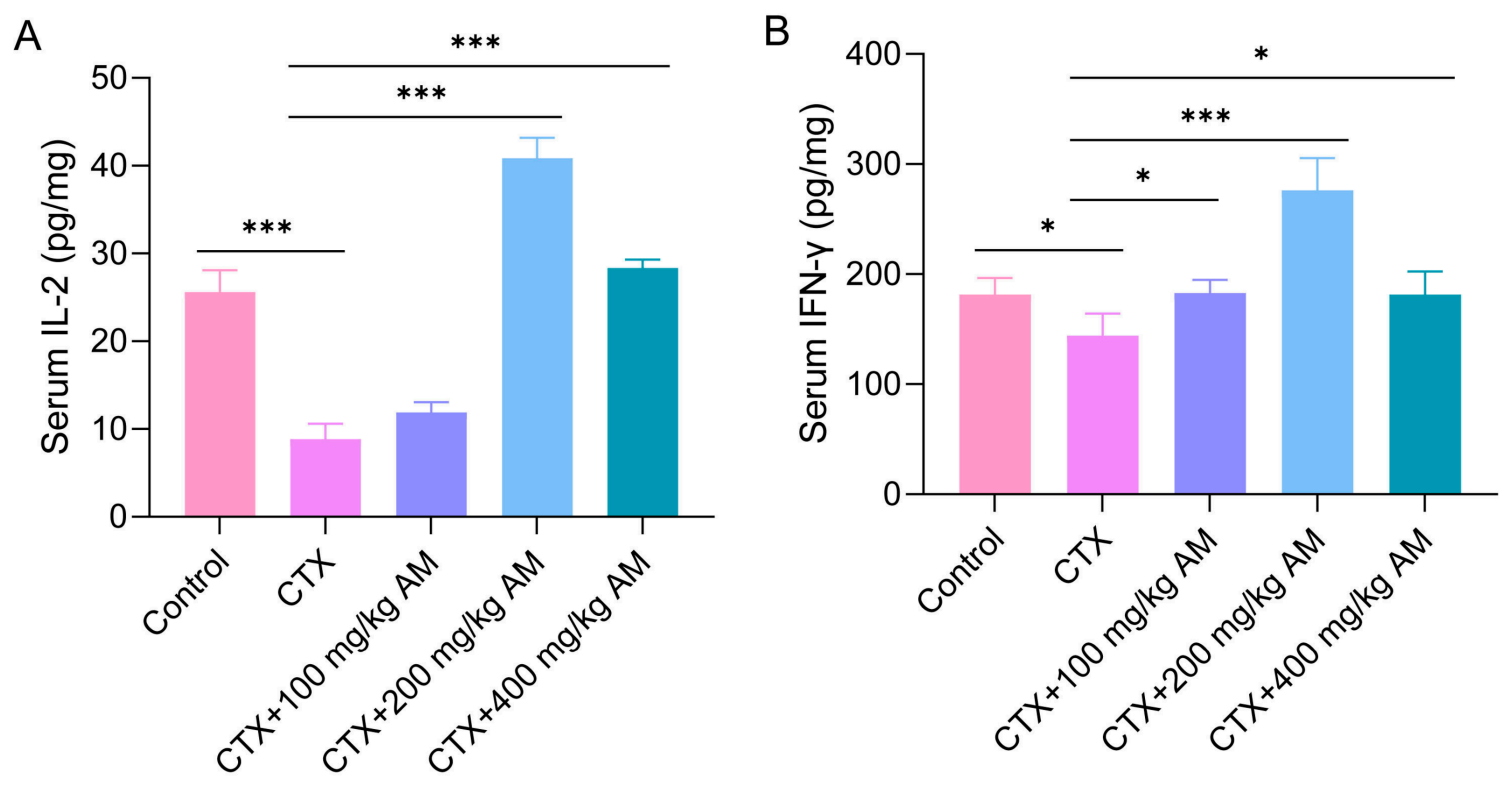
AM extract modulates serum cytokine levels in immunosuppressed mice. (**A**,**B**) Serum levels of cytokines IL-2 (**A**) and IFN-γ (**B**) measured via ELISA. Data are mean ± SD (*n* = 3). Statistical significance was determined by one-way ANOVA followed by Tukey’s post hoc test. * *p* < 0.05, *** *p* < 0.001.

Flow cytometry analysis was performed to examine the changes in CD4^+^ and CD8^+^ T cells in the spleen (Figure S1). Compared to the control group, the percentage of splenic CD4^+^ and CD8^+^ T cells in the model group was significantly decreased (Figure S1C,D, *p* < 0.01). The proportions of CD4^+^ and CD8^+^ T cells in the 200 and 400 mg/kg AM-treated groups were higher than those in the CTX model group (Figure S1C,D, *p* < 0.01), while no significant difference was observed in the 100 mg/kg AM group. Serum levels of IL-2 and IFN-γ were determined using an ELISA assay. Compared with the control group, serum IL-2 (Figure 3A, *p* < 0.001) and IFN-γ (Figure 3B, *p* < 0.05) levels in the CTX model group were significantly reduced, consistent with an immunosuppressive state. Compared with the model group, AM extract administration significantly elevated IL-2 levels (Figure 3A, *p* < 0.001 for CTX vs. CTX + 200 and CTX + 400 mg/kg AM,) and IFN-γ levels (Figure 3B, *p* < 0.05 for CTX vs. CTX + 100 and CTX + 400 mg/kg AM; *p* < 0.001 for CTX vs. CTX + 200 mg/kg AM). These results indicated that AM extract treatment increases CD4^+^/CD8^+^ T cells and enhances serum cytokine production.

### 2.4. AM Extract Enhances Vaccine-Elicited T Cell Functional Recall Responses

**Figure 4:**
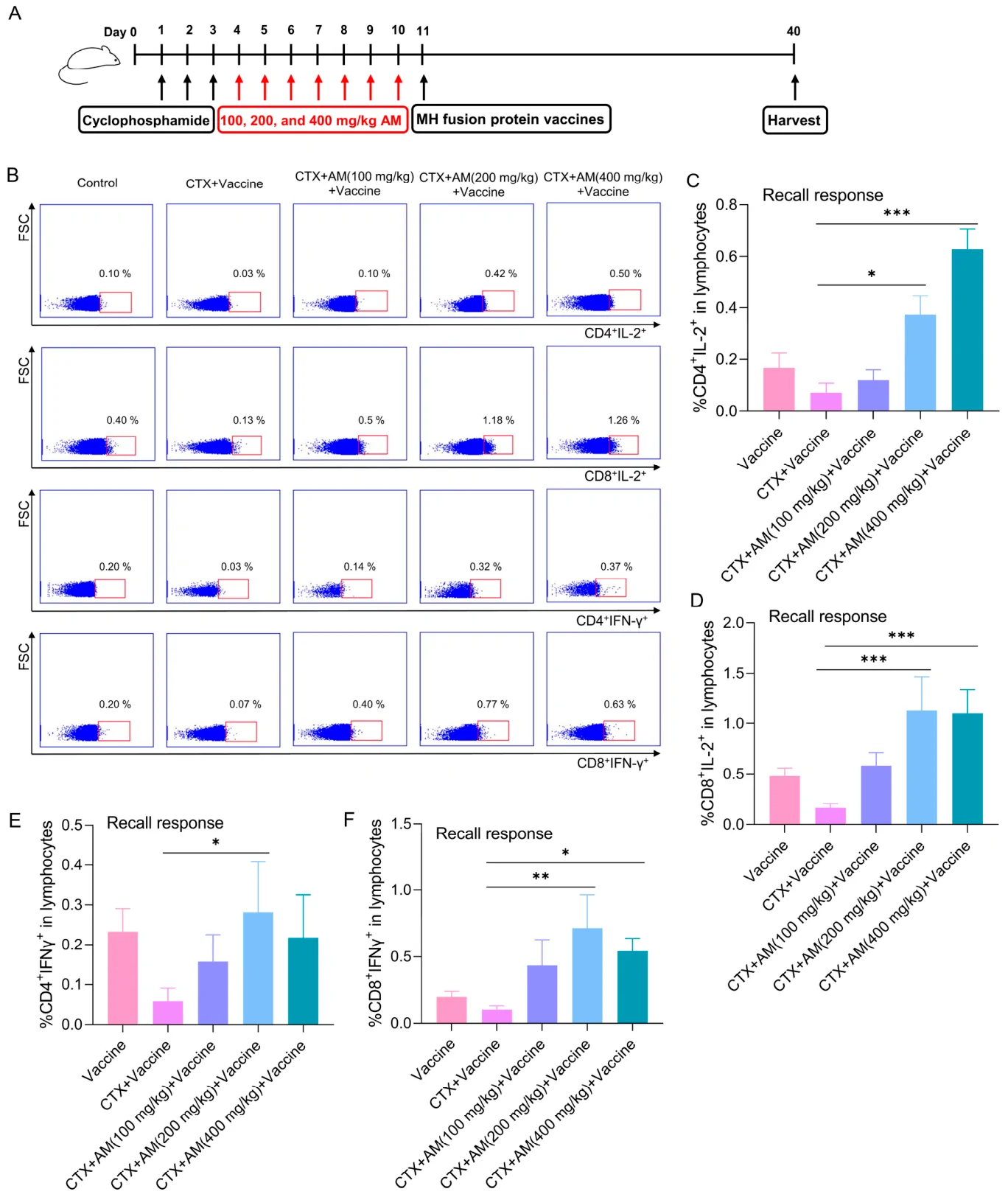
AM extract enhances antigen-specific cytokine production in T cells of vaccinated, immunosuppressed mice. (**A**) Diagram of experimental schedule. Immunosuppressed mice were treated with AM for 7 days prior to subcutaneous immunization with the subunit vaccination. (**B**) Representative flow cytometry plots showing IL-2 and IFN-γ production in gated splenic CD4^+^ and CD8^+^ T cells after in vitro HspX stimulation. (**C**) Frequencies of IL-2^+^ CD4^+^ T cells. (**D**) Frequencies of IL-2^+^ CD8^+^ T cells. (**E**) Frequencies of IFN-γ^+^ CD4^+^ T cells. (**F**) Frequencies of IFN-γ^+^ CD8^+^ T cells. Data are mean ± SD (*n* = 4). Statistical significance was determined by one-way ANOVA followed by Tukey’s post hoc test. * *p* < 0.05, ** *p* < 0.01, *** *p* < 0.001.

After 7 consecutive days of AM treatment, CTX-induced immunosuppressed mice were subcutaneously (s.c.) injected with a tuberculosis subunit vaccine to induce the antigen-specific functional recall responses of T cells (Figure 4A). After ex vivo stimulation with HspX antigen (5 μg/mL), recall responses were assessed by quantifying antigen-specific cytokine production in both CD4^+^ and CD8^+^ T cells. Compared with the CTX + Vaccine group, the frequencies of IL-2^+^ and IFN-γ^+^ CD4^+^ T cells were significantly increased in the CTX + AM (200 mg/kg) + Vaccine group (Figure 4B,C,E, *p* < 0.05). Similarly, the frequency of IL-2^+^ CD4^+^ T cells was also significantly elevated in the CTX + AM (400 mg/kg) + Vaccine group relative to the CTX + Vaccine group (Figure 4B,C, *p* < 0.001). The CTX + AM (100 mg/kg) +Vaccine group showed an increasing trend, but no statistically significant difference was observed. In addition, the proportions of IL-2^+^ and IFN-γ^+^ CD8^+^ T cells in the CTX + AM (200 mg/kg) + Vaccine group (both *p* < 0.001 for IL-2^+^; *p* < 0.01 for IFN-γ^+^) and the CTX + AM (400 mg/kg) + Vaccine group (both *p* < 0.001 for IL-2^+^; *p* < 0.05 for IFN-γ^+^) were significantly increased compared with the CTX + Vaccine group (Figure 4B,D,F). Of the three AM extract doses evaluated, 200 mg/kg elicited the most consistent augmentation of antigen-specific CD8^+^ T cell cytokine recall responses, while the 400 mg/kg dose failed to confer any additional enhancement. This optimal dose was consequently chosen for subsequent downstream molecular analyses.

### 2.5. AM Extract Remodels DNA Methylation and Cpt1a Expression in CD8^+^ T Cells

**Figure 5:**
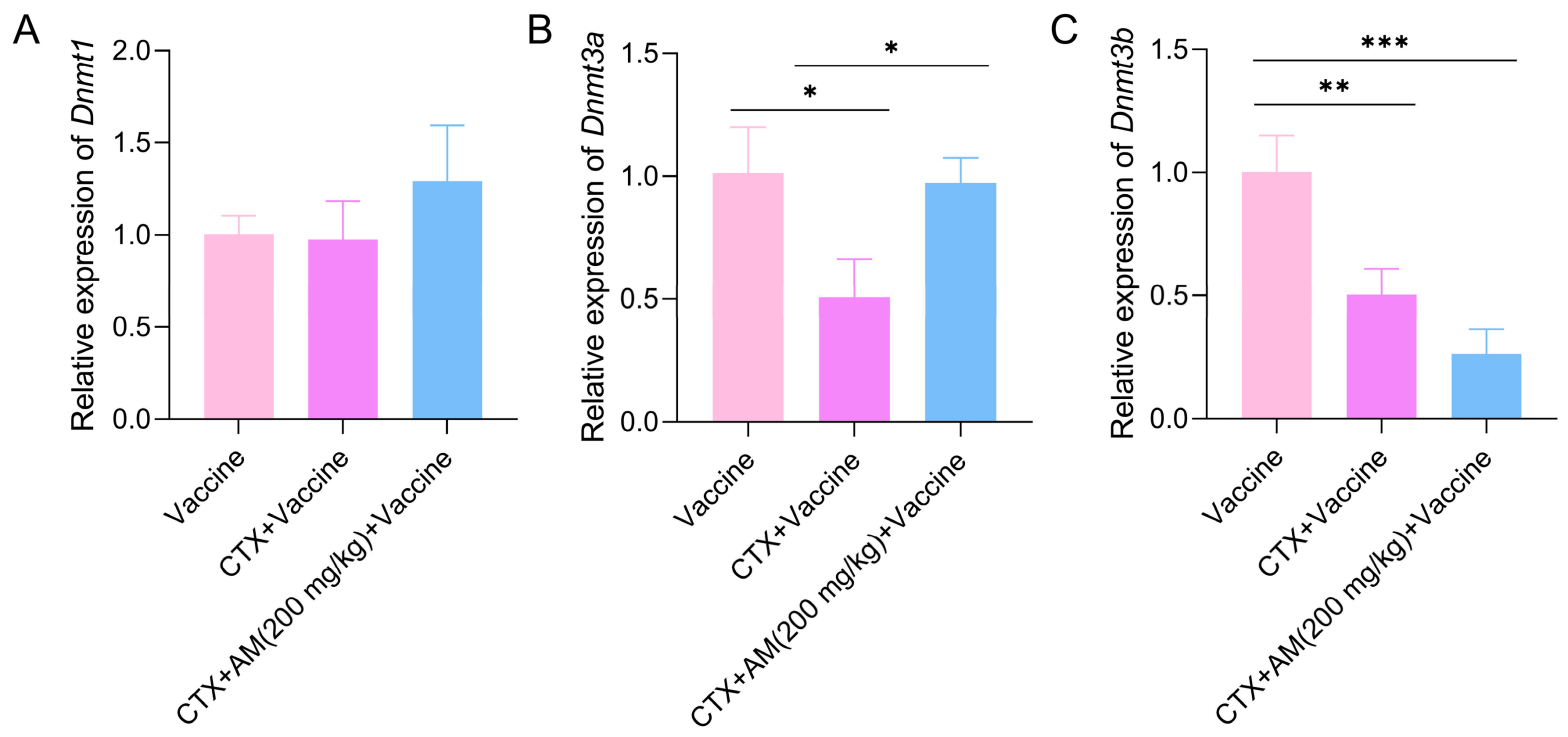
AM reverses CTX-induced downregulation of *Dnmt3a* in CD8^+^ T cells. (**A**–**C**) Relative mRNA expression levels of (**A**) *Dnmt1*, (**B**) *Dnmt3a* and (**C**) *Dnmt3b* in CD8^+^ T cells. Data are mean ± SD (*n* = 4). Statistical significance was determined by one-way ANOVA followed by Tukey’s post hoc test. * *p* < 0.05, ** *p* < 0.01, *** *p* < 0.001.

Considering the established regulatory effects of epigenetic modulation on T cell function, we further explored whether DNA methylation contributes to AM-induced changes in *Cpt1a* expression in CD8^+^ T cells post vaccination. Compared with the Vaccine group, *Dnmt3a* mRNA expression was significantly downregulated in the CTX + Vaccine group. Moreover, 200 mg/kg AM co-treatment increased its expression to a level significantly higher than that of the CTX + Vaccine group (Figure 5B, *p* < 0.05). In contrast, no significant differences in *Dnmt1* and *Dnmt3b* expression were observed between the CTX + Vaccine and CTX + AM (200 mg/kg) + Vaccine groups (Figure 5A,C), while *Dnmt3b* expression was significantly reduced in both CTX-containing groups compared with the Vaccine group.

To further identify the potential active constituent in AM extract that directly interacts with DNMT3A, molecular docking analysis was performed to screen 8 major AM components against DNMT3A (Figure S2). Astragaloside IV showed the highest docking score (−7.9 kcal/mol) (Table S2), although the values of all tested compounds fell within a narrow range (−6.9 to −7.9 kcal/mol; Figure S2A,B).

**Figure 6:**
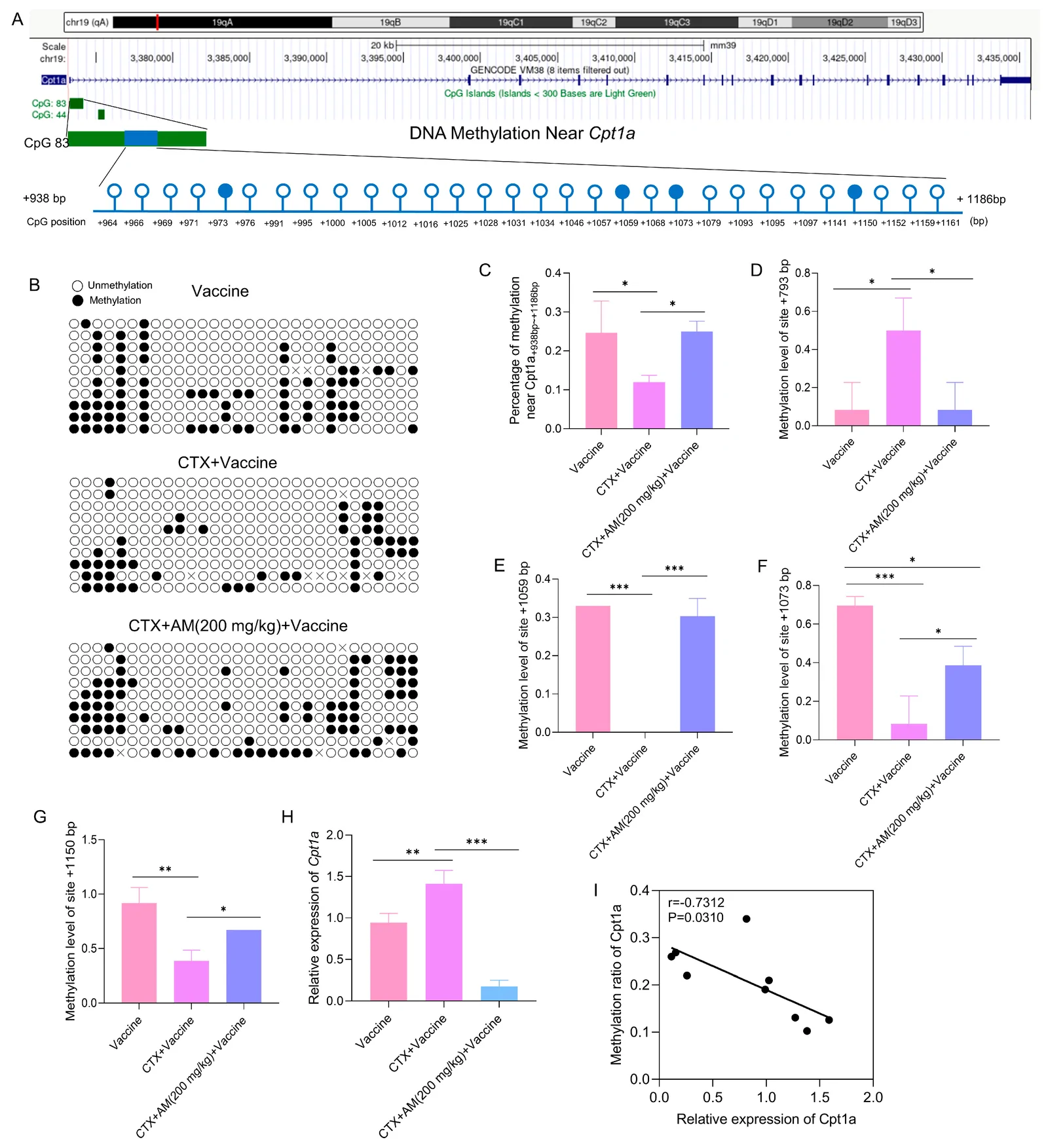
AM extract restores DNA methylation near the *Cpt1a* in CD8^+^ T cells of vaccinated, immunosuppressed mice. (**A**) Distribution of CpG sites near the *Cpt1a* gene region. CpG sites are numbered relative to the transcription start site (TSS). (**B**) Methylation patterns of individual clones determined by bisulfite sequencing. Each row represents a single clone; each column represents a CpG site. Open and filled circles indicate unmethylated and methylated cytosines, respectively. (**C**) The statistical graph of methylated ratio of *Cpt1a* in CD8^+^ T cells from the Vaccine, CTX + Vaccine and CTX + 200 mg/kg AM + Vaccine treatment groups. (**D**–**G**) Methylation levels at four specific CpG sites located at +793 bp (**D**), +1059 bp (**E**), +1073 bp (**F**), and +1150 bp (**G**) downstream of the TSS. (**H**) Relative expression levels of *Cpt1a*. (**I**) Correlation analysis between *Cpt1a* mRNA expression and its methylation level. Data are mean ± SD (*n* = 3). Statistical significance was determined by one-way ANOVA followed by Tukey’s post hoc test. * *p* < 0.05, ** *p* < 0.01, *** *p* < 0.001.

To investigate whether the observed *Dnmt3a* upregulation was accompanied by altered DNA methylation at the *Cpt1a* locus, bisulfite sequencing was conducted on a 30-site CpG island proximal to the *Cpt1a* transcription start site (Figure 6A). Compared with the Vaccine group, CTX + Vaccine treatment significantly reduced the average methylation levels of this CpG island (Figure 6B,C, *p* < 0.05). AM treatment improved the average methylation levels of this CpG island to a state comparable to that of the Vaccine group (Figure 6B,C, *p* > 0.05 vs. the Vaccine group, no statistically significant difference). Analysis of individual CpG sites revealed that CTX + Vaccine treatment led to significant hypomethylation at three sites (+1059 bp, +1073 bp, and +1150 bp relative to the TSS) compared with the Vaccine group, while the +793 bp site exhibited hypermethylation (Figure 6D–H, *p* < 0.001 at +1059 bp; *p* < 0.001 at +1073 bp; *p* < 0.01 at +1150 bp; *p* < 0.05 at +793 bp). AM extract treatment reversed the methylation alterations at all four CpG sites, restoring the methylation levels of three sites (+793, +1059, and +1150) to levels comparable to those in the Vaccine group (Figure 6D,E,G, *p* > 0.05 vs. the Vaccine group). At the +1073 site, AM extract treatment significantly increased the methylation level compared with the CTX + Vaccine group, although it did not fully increase the methylation status to the level observed in the Vaccine group (Figure 6F, *p* < 0.05). Next, we detected *Cpt1a* mRNA expression in CD8^+^ T cells. The expression of *Cpt1a* in the CTX + Vaccine group was significantly higher than that in the Vaccine group (Figure 6H, *p* < 0.01). AM extract treatment reversed this upregulation, as *Cpt1a* expression in the CTX + AM (200 mg/kg) + Vaccine group was significantly lower than that in the CTX + Vaccine group (Figure 6H, *p* < 0.001). Moreover, the AM-induced DNA methylation changes near *Cpt1a* were negatively correlated with its mRNA expression (Figure 6I, *p* < 0.05). In addition, the change trend of CPT1A protein in CD8^+^ T cells was consistent with its mRNA patterns, although the difference did not reach statistical significance (Figure S3).

## 3. Discussion

The study demonstrates that crude AM root extract can enhance immune function in CTX-induced immunosuppressed mice. This immunopotentiating effect is reflected as alleviated structural damage of spleen, significantly elevated frequencies of splenic CD4^+^ and CD8^+^ T cells and serum cytokine levels, as well as enhanced recall response of Hspx-specific CD8^+^ T cells. Such immune improvement is linked to the epigenetic regulation of the *Cpt1a* gene in CD8^+^ T cells. Mechanistically, the downregulation of *Cpt1a* expression induced by AM extract occurs concurrently with hypermethylation at the *Cpt1a* locus. Therefore, the correlation between *Cpt1a* and AM-mediated immune improvement suggests that *Cpt1a* may act as a key epigenetic node involved in this process.

Chemotherapy-induced immunosuppression is a critical unaddressed clinical challenge, as this pathological state not only disrupts multiple aspects of host immune function, but also increases patients’ susceptibility to life-threatening infections. Given the clinical limitations of existing synthetic immunomodulators, developing safe, well-tolerated natural products to restore impaired immune function has become a major translational strategy to advance comprehensive cancer therapy. AM is a traditional Chinese herbal medicine widely used to alleviate fatigue and enhance host defense, and it has been reported to promote lymphocyte proliferation, regulate cytokine production, and augment macrophage phagocytosis [21]. These multifaceted immunoregulatory properties make AM as a highly valuable candidate for developing novel interventions to accelerate immune recovery in chemotherapy-treated patients.

The spleen and thymus are primary sites for lymphocyte maturation and immune activation, and their structural integrity is critical for effective immune reconstitution following immunosuppressive insult [22]. In this study, AM extract alleviated CTX-induced spleen and thymus atrophy [23,24], and increased splenic CD4^+^ and CD8^+^ T cells populations [25]. This result suggests that the immune enhancement of AM extract may be linked to the recovery of the lymphoid microenvironment required for T cell development. Further serum cytokine assays showed that the rebound of serum IL-2 and IFN-γ in the AM extract group was trend-parallel to the recovery of splenic T cell populations. This finding supports the reasonable speculation that AM extract can effectively promote the functional recovery of type 1 cytokine secretion. Previous studies have confirmed that IL-2 is essential for T cell proliferation and survival, while IFN-γ effectively drives Th1 and cytotoxic T cell responses [26,27,28,29,30]. The two cytokines jointly provide necessary proliferative signals and functional support for the restored T cell pool. Of note, the serum levels of IL-2 and IFN-γ in the 200 mg/kg AM group were higher than those in the 400 mg/kg AM group in this study. This typical non-linear dose–response is consistent with published research conclusions. Previous studies have confirmed that purified AM polysaccharides exhibit optimal immunomodulatory activity at approximately 300 mg/kg in mice, while both lower and higher doses only produce submaximal effects [31]. In addition, previous in vivo experiments have clearly verified that when the dose of AM extract is further increased to 3.0 g/kg crude drug, it will downregulate rather than upregulate the levels of IFN and IL-1 [32]. Oral AM extract administration in human subjects induces transient elevations of IFN-γ, TNF-α and soluble IL-2R, all returning to baseline within 24h [33]. This confirms AM modulates cytokines via homeostatic feedback, not progressive dose-dependent accumulation. Combining the results of this study and published data, the 200 mg/kg dose falls exactly within the effective optimal window for driving Th1-type cytokine production in the immunosuppressive pathological environment. These findings position AM root extract as an immunomodulator that rebuilds immune homeostasis rather than a common immunostimulant that only non-specifically amplifies the overall immune response.

Upon antigen re-stimulation, memory T cells secrete cytokines to mediate immune crosstalk and functional regulation, forming the core mechanistic basis of their effector functions [26,34,35]. In the immunosuppression model of this study, 200 mg/kg AM extract intervention significantly increased the frequency of antigen-specific CD4^+^ and CD8^+^ T cells capable of producing IL-2 and IFN-γ, compared with the control group treated only with cyclophosphamide plus vaccine. It demonstrates that AM extract can effectively enhance the host’s functional antigen-specific recall response under chemotherapy-induced immunosuppressive conditions. Notably, the recovery of the CD8^+^ T cell population was more pronounced, and both 200 and 400 mg/kg AM treatments induced significant upregulation of cytokine production in this subset. Increased production of IL-2 and IFN-γ is critical for recall responses, as IL-2 drives clonal expansion required for memory T cell maintenance, while IFN-γ mediates the effector phase of secondary responses [26,27]. The elevated levels of these two cytokines indicate that AM not only supports the proliferative capacity but also preserves the effector function of re-activated T cell populations. This finding further suggests that CD8^+^ T cells may be more sensitive to AM-mediated immunomodulation, enabling them to rapidly expand and acquire effector functions upon re-exposure to antigen.

DNA methylation, as a stable epigenetic mechanism, links environmental changes to the regulation of gene expression [36,37]. To determine whether DNA methylation is involved in AM-mediated immunomodulation, we first detected the expression of DNA methyltransferases in CD8^+^ T cells after vaccination. Among *Dnmt1*, *Dnmt3a* and *Dnmt3b*, only *Dnmt3a* exhibited significant differential expression between the CTX + Vaccine group and the CTX + 200 mg/kg AM + Vaccine group. Its expression was reduced by CTX treatment and increased by AM co-administration. Molecular docking prediction indicates that astragaloside IV, a core component of AM extract, is a potential DNMT3A-binding compound that can modulate its enzymatic activity. Although this in silico finding requires further experimental validation, it provides a rational starting point for future related studies. Given the established role of *Dnmt3a* in de novo DNA methylation and the critical importance of fatty acid oxidation in CD8^+^ T cell memory formation [38,39], DNA methylation alterations were detected at the *Cpt1a* locus in CD8^+^ T cells. This analysis was inspired by previous reports that *Dnmt3a* is involved in the methylation of the *Cpt1a* promoter in neural progenitor cells [40]. The bisulfite sequencing was performed on a 30-CpG island near the *Cpt1a* transcription start site in CD8^+^ T cells to further verify this regulatory effect. CTX-induced immunosuppression triggered global hypomethylation in this region, and AM restored this chemotherapy-related epigenetic abnormality to the level of the CTX-free vaccine group. Notably, this study observed that CTX induced bidirectional heterogeneous CpG methylation alterations at the *Cpt1a* locus, and AM exerted significant regulatory effects on these four abnormal sites. The regulatory effect that precisely restored 3 sites to the baseline level of the vaccine group provides a novel low-toxic intervention strategy for epigenetic homeostasis repair under immunosuppressive conditions. Crucially, the above-baseline hypermethylation feature at the +1073 site indicates that AM does not merely passively antagonize CTX-induced epigenetic damage. Instead, it may actively recruit specific methylation modification complexes to perform targeted enhanced modification on this functional site. These unique site-selective regulatory patterns provide potential research targets for subsequent elucidation of the mechanism by which *Cpt1a* epigenetic modification remodels the fatty acid oxidation pathway in CD8^+^ T cells.

A core question to be clarified in this study is whether the aforementioned methylation changes in CD8^+^ T cells after vaccination directly affect *Cpt1a* gene expression. Our results show that CTX + Vaccine treatment significantly upregulates *Cpt1a* mRNA levels, while AM intervention reverses this trend and suppresses its aberrant overexpression. *Cpt1a* methylation level is significantly negatively correlated with its mRNA expression, which is consistent with the classic epigenetic mechanism of “DNA methylation mediating transcriptional repression”. CPT1A protein expression trend is consistent with mRNA data, though no statistical significance is reached due to the limited sample size, and further validation with a larger cohort will consolidate this conclusion. Notably, the regulatory pattern of *Cpt1a* observed in this study seems to be inconsistent with the well-recognized role of FAO in CD8^+^ T cell memory formation established paradigm that FAO supports the long-term survival of memory T cells [17,39]. However, while FAO is established to support memory T cell survival, emerging evidence suggests that aberrant CPT1a-driven FAO may contribute to CD8^+^ T cell metabolic rigidity and functional impairment. In models of T cell exhaustion, *Cpt1a* is upregulated as a compensatory survival mechanism, yet this metabolic state remains insufficient to sustain robust effector function [41]. Furthermore, abnormal activation of fatty acid metabolism accelerates effector-to-exhaustion transitions in CD8^+^ T cells, and pharmacological inhibition of CPT1A enhances CD8^+^ T cell functionality [39,41,42]. In the present study, persistent upregulation of *Cpt1a* was observed in CTX-immunosuppressed CD8^+^ T cells, which may represent a similar state of metabolic maladaptation. Under these circumstances, AM-mediated suppression of *Cpt1a* may relieve this metabolic burden, thereby allowing T cells to redirect resources toward long-term effector functions rather than mere survival. This interpretation aligns with our observation that CTX-induced aberrant *Cpt1a* upregulation is accompanied by impaired cytokine production in CD8^+^ T cells, while AM-mediated *Cpt1a* downregulation coincides with the recovery of T cell effector functions. These correlative findings position *Cpt1a* as a candidate key target of AM-mediated epigenetic regulation in CD8^+^ T cells, providing a preliminary mechanistic framework for understanding how AM restores adaptive immunity after chemotherapy.

Several methodological limitations of this study should be explicitly acknowledged. Without a dedicated AM-only group in immunocompetent mice, it remains unclear if the effects stem from AM rescuing CTX-induced epigenetic dysregulation or from AM independently modulating global methylation. While the consistent normalization of all CTX-disrupted parameters to the vaccine baseline supports a context-dependent rescue effect, a dedicated AM-only control group with parallel assessments is needed in future studies to distinguish between those two mechanisms. Two technical limitations should be noted: Fc receptor blocking and viability dye exclusion were not included in the flow cytometry workflow. However, potential artifacts were largely mitigated by re-compensation and standardized gating, and the resulting trends were fully consistent with the cytokine and histological findings, with data reliability validated via cross-reference. This study did not complete full phenotypic characterization of memory T cell subsets using the canonical CD44 and CD62L markers, nor did it perform gain- or loss-of-function experiments to establish direct causal inference for the *Dnmt3a*–*Cpt1a* axis. Future studies integrating these complementary approaches will be critical to fully delineate the complete mechanistic framework underlying AM crude extract-mediated immune restoration after chemotherapy.

## 4. Materials and Methods

### 4.1. Materials

*Astragalus membranaceus* (AM) (5-year-old, collected from Longxi County, Gansu Province, China in October 2024) was authenticated by Prof. Mengfei Li (College of Agronomy, Gansu Agricultural University). Dried AM roots (500 g) were powdered, boiled in 5 L distilled water for 2 h, and filtered through gauze. The residue was re-extracted once under the same conditions, and the combined filtrates yielded the crude AM extract. The pooled filtrates were concentrated to a relative density of 1.025 g/mL (crude drug equivalent) using a rotary evaporator and stored at 4 °C for subsequent use. This crude aqueous extract was selected on the grounds that water decoction, as a conventionally used preparation method in clinical practice, is capable of fully retaining water-soluble immunomodulatory components that are closely linked to vaccine-induced immune responses.

Subunit vaccine and single antigen preparation. The Mtb10.4-HspX (MH) fusion protein vaccines were formulated following an established protocol [43]. Each 0.2 mL dose contained 5 µg of the MH antigen, combined with an adjuvant system consisting of 250 µg of DDA (N,N′-dimethyl-N,N′-dioctadecylammonium bromide) (Sigma-Aldrich, Poole, UK) and 50 µg of poly(I:C) (polyinosinic–polycytidylic acid) (Sigma-Aldrich, Poole, UK). The recombinant HspX antigen was purified according to established methods using Ni-NTA His affinity chromatography [43,44].

### 4.2. Chemical Composition Analysis of AM Based on UPLC-MS

To characterize the chemical profile of the AM extract, an aliquot of the lyophilized decoction (equivalent to 1.0 g crude drug) was dissolved in 20 mL of 70% methanol, sonicated (250 W, 40 kHz) for 40 min, and centrifuged. The supernatant was filtered through a 0.22 μm PTFE membrane prior to UPLC-MS analysis. Three biological replicates were prepared independently. Chromatographic separation was performed on an Agilent Eclipse Plus C18 column (100 mm × 4.6 mm, 1.8 μm, Agilent Technologies, Santa Clara, CA, USA) at 30 °C. The mobile phase consisted of 0.1% formic acid in water (A) and 0.1% formic acid in acetonitrile (B) at a flow rate of 0.3 mL/min. The gradient was: 0–5 min, 5% B; 5–10 min, 5–30% B; 10–30 min, 30–35% B; 30–35 min, 35–50% B; 35–40 min, 50–80% B; 40–50 min, 80–95% B; followed by 5 min re-equilibration at 5% B. Injection volume was 2 μL. Mass spectrometry was performed using a HESI source in both positive and negative ionization modes. Key parameters: spray voltage, +3.5 kV and −3.0 kV; sheath gas, 35 arb; auxiliary gas, 10 arb; capillary temperature, 320 °C; full scan resolution, 70,000; dd-MS^2^ resolution, 17,500; scan range, *m*/*z* 100–1500; stepped NCE, 20, 40, 60 eV. Data were processed using Xcalibur 2.0 and Compound Discoverer 3.0. Metabolites were putatively identified by matching accurate mass (±5 ppm), MS/MS fragmentation patterns, and retention times against the ChemSpider, mzCloud, and mzVault databases. Identification confidence levels were assigned according to the metabolomics standards initiative guidelines.

### 4.3. Animal and Treatment

Female C57BL/6J mice (6 weeks old, 18–22 g) were obtained from the Animal Center of the Lanzhou Veterinary Research Institute, Chinese Academy of Agricultural Sciences. All procedures were approved by the Animal Care and Use Committee of Gansu Agricultural University (Approval No. GSAU-Eth-LST-2024-012) on 12 March 2024. Following a one-week adaptation, mice were randomly assigned to five groups (*n* = 12 per group): (1) Control; (2) CTX-induced immunosuppression (CTX); (3) CTX + 100 mg/kg AM; (4) CTX + 200 mg/kg AM; (5) CTX + 400 mg/kg AM. The immunosuppression model was established via daily intraperitoneal (i.p.) injection of CTX at a dose of 100 mg/kg for 3 consecutive days (days 1–3). From day 4, mice in different treatment groups were administered AM crude extract at graded doses of 100, 200, or 400 mg/kg body weight via daily oral gavage daily for 7 consecutive days. Control and CTX groups received an equal volume of distilled water. Body weight was recorded daily.

On day 11 post-treatment, six mice from each group were euthanized for subsequent assessments, including immune organ index calculation, histopathological analysis, serum cytokine detection, and T cell subset analysis. The remaining six mice in the Control group, CTX group, and AM-treated groups (100, 200, and 400 mg/kg) received subcutaneous (s.c.) immunization with the tuberculosis subunit vaccine (200 μL/mouse). No further AM administration was given after vaccination. All immunized mice were euthanized on day 40 (one month post-immunization) for immune response evaluation. For subsequent molecular analyses (CD8^+^ T cell sorting, RNA/DNA extraction, RT-qPCR, and bisulfite sequencing), three groups were selected, namely Vaccine (Control), CTX + Vaccine, and CTX + AM (200 mg/kg) + Vaccine.

### 4.4. Immune Organ Index Detection

The thymus and spleen were carefully dissected and weighed. Organ indices were calculated as follows [45]: thymus index (mg/g) = thymus weight (mg)/body weight (g); spleen index (mg/g) = spleen weight (mg)/body weight (g).

### 4.5. Histopathological Analysis

Spleen tissues were fixed in 4% paraformaldehyde, embedded in paraffin, sectioned at 4 μm, and stained with hematoxylin and eosin (H&E) (Solarbio, Beijing, China). Histopathological changes were evaluated under a light microscope (AxioScope A1, Carl Zeiss AG, Oberkochen, Germany). Splenic architecture, including white pulp area and germinal center formation, was semi-quantitatively scored on a 0–3 scale by two blinded pathologists (Table S1).

### 4.6. Detection of Serum Cytokines in Serum

Blood samples were centrifuged at 3500 rpm for 15 min at 4 °C to obtain serum. A few serum samples were excluded due to hemolysis, resulting in *n* = 3 per group for cytokine analysis. Serum levels of IL-2 and IFN-γ were quantified using commercial ELISA kits (Solarbio, Beijing, China) according to the manufacturer’s protocol. Each sample was measured in duplicate, and absorbance was read at 450 nm with a correction wavelength of 570 nm.

### 4.7. Flow Cytometric Analysis

On day 11, spleens were harvested from 4 mice per group (*n* = 4), and lymphocytes were isolated using lymphocyte separation medium (Dakewe Biotech Co., Ltd., Shenzhen, China) following the manufacturer’s instructions. Fresh spleens were gently homogenized in separation medium (≤5 min), centrifuged at 500× *g* for 20 min at RT, and the interface lymphocytes were collected. The cells were washed twice with PBS (250 g, 10 min) to obtain purified splenic lymphocytes. For CD4^+^ and CD8^+^ T cell analysis, isolated cells were stained with anti-CD3-PE (17A2, eBioscience, San Diego, CA, USA), anti-CD4-FITC (RM4-5, eBioscience) and anti-CD8-PerCP-Cy5.5 (53–6.7, eBioscience) at 4 °C for 30 min. Samples were acquired on a BD Accuri C6 Plus flow cytometer and analyzed with the instrument software. Flow cytometry analysis was performed using a sequential gating strategy. Lymphocytes were first gated based on FSC/SSC scatter plots, followed by CD3^+^ T cell gating, and finally CD4^+^ and CD8^+^ subsets were analyzed within the CD3^+^ gate. Since no viability dye was used, dead cells were excluded by FSC/SSC gating. Uniform gating criteria were applied across all experimental groups.

On day 40, spleens were harvested from six immunized mice per group. Spleens were harvested to prepare splenic lymphocyte suspensions, and lymphocytes were subsequently isolated following the procedure described above. For T cell recall response analysis, cells from four mice per group were seeded in 24-well plates at 5 × 10^6^ cells per well in 2 mL of complete RPMI 1640 medium and stimulated with 5 μg/mL HspX antigen at 37 °C under 5% CO_2_. After 4 h of stimulation, BD GolgiPlug™ (containing brefeldin A, BD Biosciences, San Diego, CA, USA) was added, and incubation was continued for an additional 8 h (total stimulation 12 h). Subsequently, cells were collected, washed with PBS, and stained with anti-CD4-FITC and anti-CD8-PerCP-Cy5.5 at 4 °C for 30 min. After staining, cells were fixed and permeabilized using the BD Cytofix/Cytoperm kit, followed by intracellular staining with anti-IFN-γ-APC (XMG1.2) and anti-IL-2-PE (JES6-5H4). Samples were acquired on a BD Accuri C6 Plus flow cytometer. Data were analyzed using the instrument’s accompanying software. In this assay, T cells were identified by CD4/CD8 surface expression. The 4-channel BD Accuri C6 Plus flow cytometer restricted panel design precluded inclusion of a pan-T cell marker (e.g., CD3). Antigen-specific production of IFN-γ and IL-2 upon HspX stimulation confirmed the T-cell specificity of the detected cytokine response.

### 4.8. Cell Sorting, RNA Extraction and RT-qPCR

Splenic cells were isolated from harvested spleens on day 40, and CD8^+^ T cells were subsequently enriched by negative selection using magnetic beads (Cat. No. 130-104-075, Miltenyi Biotec, Bergisch Gladbach, Germany). Purity (>90%) was confirmed by flow cytometry. Sorted CD8^+^ T cell viability was not evaluated via dye exclusion. The gentle negative selection (Miltenyi Biotec) maintains high cell viability, and nucleic acids were extracted immediately post-sorting. Total RNA was extracted using the PureLink RNA Mini Kit (Cat. No. 12183018A, Thermo Fisher Scientific, Waltham, MA, USA). RNA integrity was verified by agarose gel electrophoresis. cDNA was synthesized from 1 μg of total RNA using the RevertAid First Strand cDNA Synthesis Kit (Thermo Fisher Scientific). RT-qPCR was performed using SYBR Green Master Mix (Cat. No. RR420A, Takara Bio Inc., Kusatsu, Japan) on a ForeQuant Real-Time PCR System. The thermal cycling conditions were 95 °C for 30 s, followed by 40 cycles of 95 °C for 5 s and 60 °C for 30 s. Melting curve analysis was performed to confirm primer specificity. Relative mRNA expression was calculated using the 2^^(–ΔΔCt)^ method, with β-actin as the reference gene. The primer sequences used in this study were listed below: *β-actin*: forward 5′-ATTCCACCCATGGCAAATTC-3′, reverse 5′-GGATCTCGCTCCTGCAAGATG-3′; *Cpt1a*: forward 5′-CTACATCACCCCAACCCATATT-3′, reverse 5′-GATCCCAGAAGACGAATAGGTT-3′; *Dnmt1*: forward 5′-GAAAAGGAGTGTGTGAGGGAGA-3′, reverse 5′-AGTGTGTGTTCCGTTCTCCAA-3′; *Dnmt3a*: forward 5′-CAGGAGAGGGCAAAGAACAGA-3′, reverse 5′-CACTCATCCCGTTTCCGTTTG-3′; *Dnmt3b*: forward 5′-ATGACTCTGCTGCTTCTG-3′, reverse 5′-CCTTGTTGTTGGTGACTTC-3′.

### 4.9. Western Blot Analysis

Total protein was extracted from isolated CD8^+^ T cells, and protein concentration was determined using a BCA Protein Assay kit (Cat. No. P0012, Beyotime Biotechnology, Shanghai, China). Subsequently, sodium dodecyl sulfate–polyacrylamide gel electrophoresis was performed with 20 μg protein loaded per well. The membranes were incubated overnight at 4 °C with primary antibody CPT1A (1:1000, Cat No. DF12004, Affinity Biosciences Ltd., Changzhou, China) and β-actin (1:5000, Cat No. 4967S, Cell Signaling Technology, Danvers, MA, USA). After incubation with secondary antibodies for 2 h, protein levels were detected using enhanced chemiluminescence reagents, and band intensities were quantified with ImageJ 1.54.

### 4.10. Molecular Docking

Molecular docking was performed between 8 major active components of AM and DNMT3A (PDB ID: 8BA5). The 3D structure of the target protein was retrieved from the Protein Data Bank (PDB), while the 3D structures of candidate ligands were downloaded from PubChem, converted to mol2 format using OpenBabel, and geometrically optimized with Chem3D 20.0.0. After the removal of water molecules and redundant extra ligands, hydrogen atoms were added and partial charges were assigned to the processed structures. The docking pocket was subsequently defined, and molecular docking simulations were performed using AutoDock Tools 1.5.7. Finally, docking outputs were visualized and analyzed with PyMOL 3.1.8.

### 4.11. DNA Extraction and Bisulfite Sequencing PCR (BSP)

Genomic DNA was extracted from sorted CD8^+^ T cells using the Genomic DNA Extraction Kit (Cat. No. D3396-01, Omega Bio-tek, Inc., Norcross, GA, USA). Sodium bisulfite conversion was performed strictly following the using manufacturer’s protocol with the BisuFlash™ DNA Modification Kit (Cat. No. P-1026-050, Epigentek Group Inc., Farmingdale, NY, USA). After sodium bisulfite treatment, unmethylated cytosines were converted to uracil, while methylated cytosines remained unchanged.

Primers targeting the region near the Cpt1a gene were designed using the Agena EpiDesigner software (Agena Bioscience, San Diego, CA, USA; http://www.epidesigner.com/start3.html, accessed on 29 December 2025), with sequences: forward 5′-GTAGTTTAGTTGGGTTTAGTTAATTATT-3′, reverse 5′-AAAATACCCTCTACTTCTCCAATTATT-3′. The 50 μL total PCR reaction system contained 5 μL bisulfite-converted DNA, 5 μL 10× EpiTaq PCR buffer, 0.5 μL TaKaRa EpiTaq HS (5 U/μL), 6 μL dNTP mixture, 5 μL forward primer and 5 μL reverse primer (10 μM), which was adjusted to 50 μL with nuclease-free water. Thermal cycling conditions were set as: initial denaturation at 95 °C for 3 min, followed by 40 cycles of 95 °C for 5 s, 52 °C for 30 s (combined annealing and extension), 72 °C for 30 s, and a final extension at 72 °C for 10 min.

PCR products were separated by agarose gel electrophoresis, purified using the PureLink™ Quick Gel Extraction Kit (Cat. No. K2100-12, Invitrogen, Carlsbad, CA, USA), and cloned into the pMD19-T vector (Cat. No. 3271, Takara Bio Inc., Kusatsu, Shiga, Japan). The ligation products were transformed into competent *Escherichia coli* cells, which were then plated on LB agar containing ampicillin, X-gal, and IPTG for blue-white screening. Ten positive (white) clones per sample were selected and sequenced. Methylation status at individual CpG sites was analyzed and visualized using the online QUMA version 1.02 (https://quma.cdb.riken.jp/, accessed on 25 August 2026).

### 4.12. Statistical Analysis

One-way ANOVA followed by Tukey’s post hoc analysis was used in this study. Normality and homogeneity of variance were confirmed prior to analysis. Data are presented as mean ± SD. A *p*-value < 0.05 was considered statistically significant. Graphs were generated using GraphPad Prism 8.0 (GraphPad Software).

## 5. Conclusions

In summary, AM root extract restores impaired adaptive immunity and enhances the functional recall response of antigen-specific CD8^+^ T cells in cyclophosphamide-induced immunosuppressive mice. At the molecular level, AM intervention is associated with upregulated *Dnmt3a* expression, increased methylation at specific sites of the *Cpt1a* gene, and downregulated *Cpt1a* expression. These coordinated changes are correlated with the observed improvement in T cell effector function. This study reveals an association between AM-mediated immune restoration and epigenetic regulation of *Cpt1a*, providing a potential mechanistic basis for its immunomodulatory effects.

## Figures and Tables

**Table 1:** Identification of chemical components in AM by UPLC-MS analysis.

NO.	tR/min	Formula	Adduct	Calculated (*m/z*)	Delta Mass ppm	Identification	Metabolite ID
1	0.764	C_4_H_7_NO_4_	[M − H]^−^	132.03023	0.03962833	L-Aspartic acid	HMDB0000191
2	0.817	C_8_H_8_O_2_	[M + H]^+^	137.05982	1.289414516	4-Hydroxy-3-methylbenzaldehyde	C21166
3	0.831	C_6_H_11_NO_2_	[M + H]^+^	130.08648	1.753449187	Pipecolic acid	HMDB0000716
4	0.855	C_12_H_21_N_3_O_6_	[M + H]^+^	304.15012	−0.642889082	Nicotianamine	C05324
5	0.869	C_6_H_14_N_4_O_2_	[M + H]^+^	175.11924	1.651593202	DL-Arginine	HMDB0251511
6	3.784	C_17_H_24_O_9_	[M − H]^−^	371.13474	−0.088064064	Syringin; Eleutheroside B	C01533
7	3.918	C_27_H_30_O_16_	[M + H]^+^	611.16088	0.358803336	Quercetin 3-O-rhamnoside 7-O-glucoside	C19796
8	4.226	C_41_H_68_O_14_	[M + H]^+^	785.46746	−0.916920158	Astragaloside IV	C17799
9	4.292	C_8_H_10_O_3_	[M − H]^−^	153.05577	116904.1915	Vanillyl alcohol	HMDB0032012
10	4.298	C_39_H_50_O_23_	[M + H]^+^	887.28412	2.882541421	Astrasikokioside I	4.298_887.28412
11	4.743	C_15_H_22_N_4_O_3_	[M + H]^+^	307.17642	−0.14688238	Feruloylagmatine	C18325
12	4.757	C_25_H_26_O_15_	[M + H]^+^	567.13483	0.640685985	Isoorientin 6″-O-alpha-L-arabinoside	4.757_567.13483
13	4.809	C_27_H_30_O_15_	[M − H]^−^	593.15124	−1.559329769	Isovitexin 2″-O-beta-D-glucoside	C04199
14	4.816	C_7_H_6_O_3_	[M − H]^−^	137.02444	0.177815477	4-Hydroxybenzoic acid	HMDB0000500
15	4.895	C_23_H_28_O_10_	[M − H]^−^	463.1605	−1.127926369	Isomucronulatol 7-O-glucoside	4.895_463.16050
16	4.92	C_47_H_78_O_19_	[M + H]^+^	947.52028	−0.658575859	Astragaloside VII	C17802
17	5.109	C_17_H_18_O_5_	[M − H]^−^	301.1073	−2.800929054	Mucronulatol	C10507
18	5.345	C_45_H_74_O_18_	[M − H]^−^	901.48332	3.413789524	Asparasaponin II	C17470
19	5.482	C_41_H_68_O_14_	[M − H]^−^	829.46096	58,648.33616	Astragaloside IV	5.482_829.46096
20	5.814	C_43_H_70_O_15_	[M − H]^−^	871.47263	55,668.50907	Astragaloside II	5.814_871.47263
21	5.988	C_43_H_70_O_15_	[M + H]^+^	827.47838	−0.439391972	Astragaloside II	C17798
22	6.072	C_47_H_76_O_17_	[M − H]^−^	911.50459	3.965776509	Soyasaponin II	C12081
23	6.094	C_48_H_78_O_18_	[M − H]^−^	941.51572	4.310669	Soyasaponin I	HMDB0034649
24	6.101	C42H68O14	[M + H]^+^	797.46774	−0.550323143	Soyasaponin III	C19865
25	6.136	C_45_H_72_O_16_	[M − H]^−^	867.47786	3.567615362	Astragaloside I	C17797
26	6.563	C_51_H_82_O_21_	[M + H]^+^	1031.5447	2.490058185	Pseudoprotodioscin	C17469

## Data Availability

The data presented in this study are available on request from the corresponding author.

## Supplementary Materials

The following supporting information can be downloaded at: https://www.mdpi.com/article/10.3390/ijms27177746/s1.

## Abbreviations

The following abbreviations are used in this manuscript:

AM	*Astragalus membranaceus*
BSP	Bisulfite sequencing PCR
CTX	Cyclophosphamide
Cpt1a	*C**arnitine palmitoyltransferase 1a*
Dnmt1	*DNA Methyltransferases 1*
Dnmt3a	*DNA Methyltransferases 3a*
Dnmt3b	*DNA Methyltransferases 3b*
FAO	Fatty acid β-oxidation
IL-2	Interleukin-2
IFN-γ	Interferon-gamma
RT-qPCR	Reverse transcription quantitative real-time PCR
SEM	Standard error of the mean
TSS	Transcription start site
UPLC-MS	Ultra-performance liquid chromatography coupled with tandem mass spectrometry
