# Supplementary material for: Astragalus membranaceus Root Extract Improves T Cell Immunity in CTX-Immunosuppressed Mice and Is Associated with Hypermethylation of the Cpt1a Locus
Source: Int J Mol Sci. 2026 Aug 29;27(17):7746. doi: 10.3390/ijms27177746 (PMC13566575; doi:10.3390/ijms27177746)
Supplement: Supplementary file 1 [file ijms-27-07746-s001.zip › ijms-4455210-supplementary.pdf]

Supplementary Materials

Table S1. Histological scores of mouse spleen pathological analysis

Scoring Dimension	Score 0 (Normal)	Score 1 (Mild)	Score 2 (Moderate)	Score 3 (Severe)
Splenic Architectural Integrity	Clear demarcation between red and white pulp; well-preserved normal tissue zoning	Slight blurring of red/white pulp boundary; no obvious structural disruption	Partially lost normal anatomical zoning; moderate disorder of splenic parenchyma	Indistinct red/white pulp boundary; severe collapse of overall splenic architecture
Vascular Congestion & Interstitial Injury	Normal splenic sinus morphology; no obvious blood stasis or necrotic foci	Focal mild vascular dilation; no evident interstitial damage	Diffuse sinus congestion accompanied by focal interstitial edema	Severe widespread vascular congestion with scattered necrotic foci in splenic parenchyma
Lymphoid Follicle (White Pulp) Atrophy	Regularly shaped lymphoid nodules with densely arranged lymphocytes	Mild shrinkage of lymphoid nodules with slightly reduced lymphocyte density	Moderate atrophy of lymphoid nodules with significantly decreased lymphocyte numbers	Severe depletion of lymphocytes with few identifiable lymphoid follicles

Table S2. Binding affinity scores of key active components of AM with core target proteins in molecular docking

Kcal/mol	Astragaloside IV	Mucronulatol	Feruloylagmatine
DNMT3A (8BA5)	-7.9	-7.0	-7.2
CPT1A (2LE3)	-6.4	-5.0	-5.4

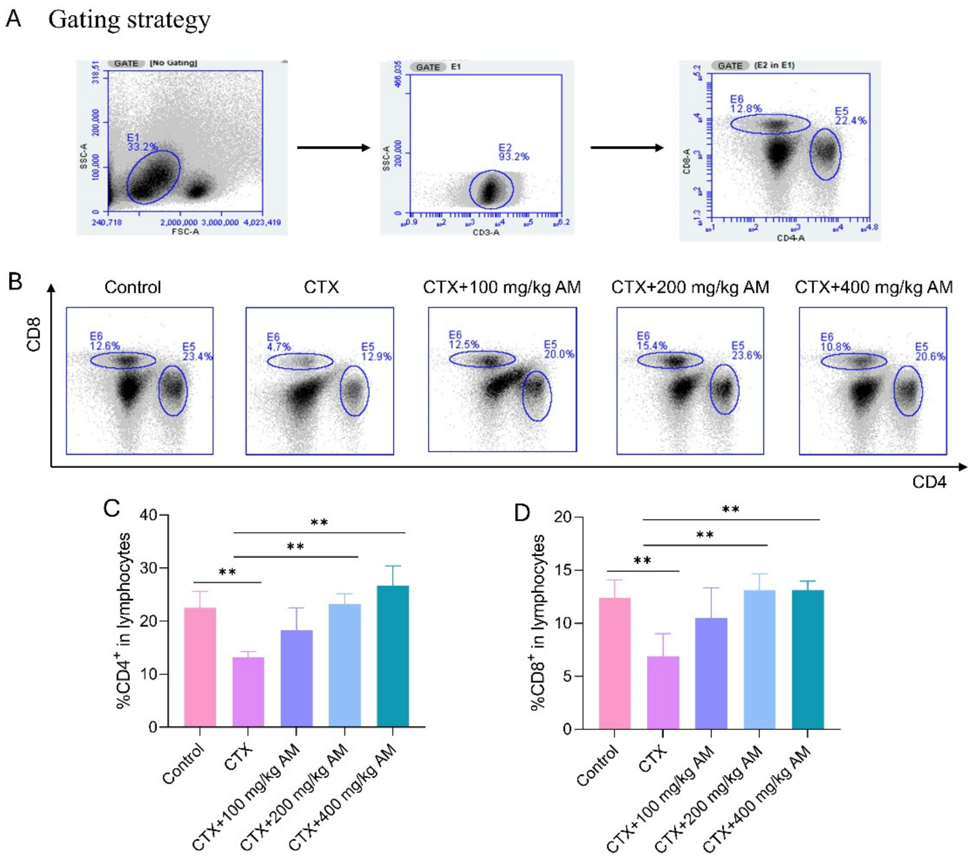

Figure S1. AM extract increased the proportion of T cell subsets in immunosuppressed mice. (A) Representative gating strategy. (B) Representative flow cytometry plots for T cell subset gating. (C-D) Percentages of splenic CD4⁺ T cells (C), CD8⁺ T cells (D). Data are mean ± SD (n = 4). Statistical significance was determined by one-way ANOVA followed by Tukey's post hoc test. $^{**}p < 0.01$.

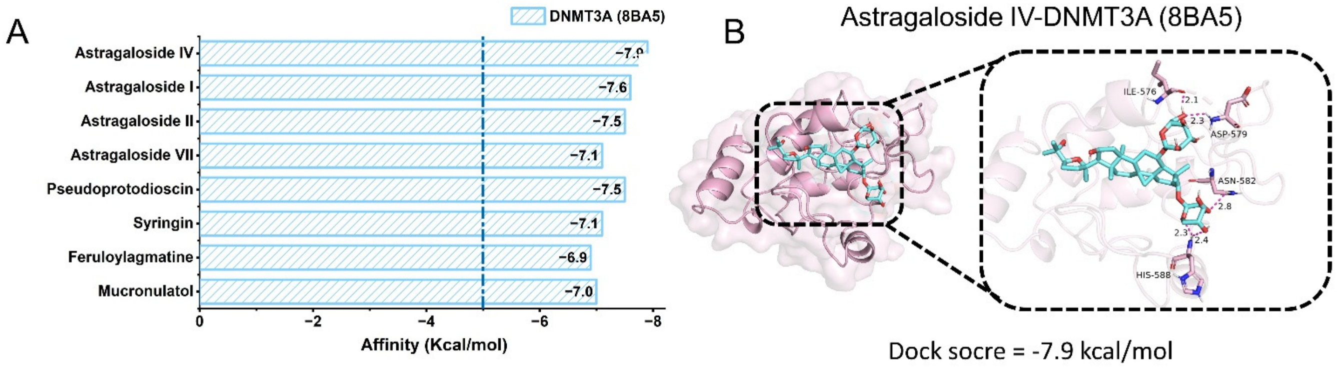

Figure S2. Molecular docking of AM components against DNMT3A. (A) Molecular docking of eight major AM components against DNMT3A. (B) The visualization of molecular docking of astragaloside IV and DNMT3A.

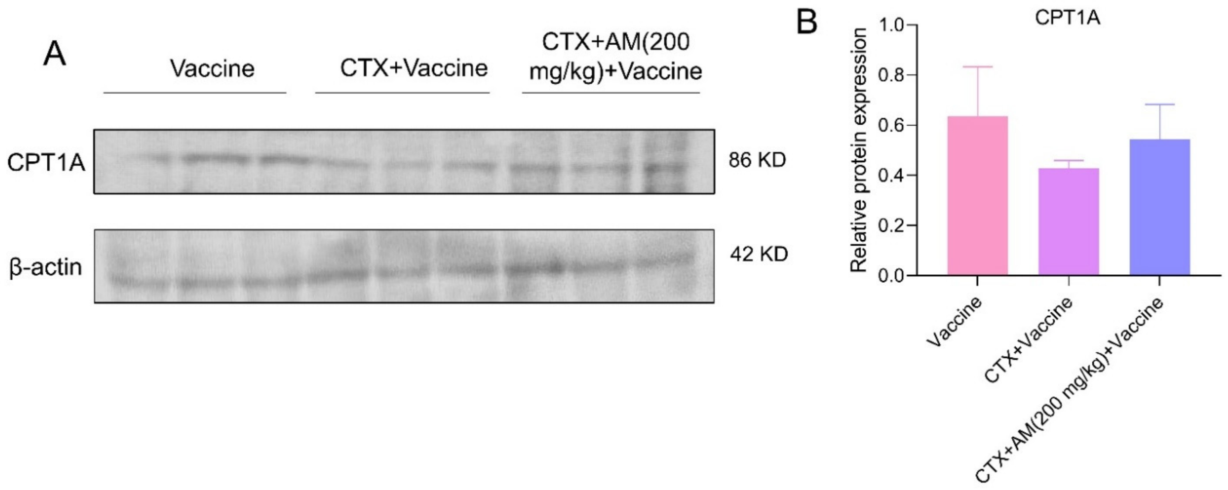

Figure S3. The protein level of CPT1A in $CD8^+$ T cells after vaccination. (A) Representative western blots showing protein levels of CPT1A; β-actin served as the loading control. (B) The relative protein expression of CPT1A. Data are mean ± SD (n = 3). Statistical significance was determined by one-way ANOVA followed by Tukey's post hoc test.
